# Proteomic Amino-Termini Profiling Reveals Targeting Information for Protein Import into Complex Plastids

**DOI:** 10.1371/journal.pone.0074483

**Published:** 2013-09-16

**Authors:** Pitter F. Huesgen, Meriem Alami, Philipp F. Lange, Leonard J. Foster, Wolfgang P. Schröder, Christopher M. Overall, Beverley R. Green

**Affiliations:** 1 Centre for Blood Research and Department of Oral Biological and Medical Sciences, University of British Columbia, Vancouver, British Columbia, Canada; 2 Department of Biochemistry and Molecular Biology, University of British Columbia, Vancouver, British Columbia, Canada; 3 Department of Botany, University of British Columbia, Vancouver, British Columbia, Canada; 4 Centre for High-Throughput Biology, University of British Columbia, Vancouver, British Columbia, Canada; 5 Department of Chemistry and Umeå Plant Science Centre, Umeå University, Umeå, Sweden; University of Padova, Italy

## Abstract

In organisms with complex plastids acquired by secondary endosymbiosis from a photosynthetic eukaryote, the majority of plastid proteins are nuclear-encoded, translated on cytoplasmic ribosomes, and guided across four membranes by a bipartite targeting sequence. In-depth understanding of this vital import process has been impeded by a lack of information about the transit peptide part of this sequence, which mediates transport across the inner three membranes. We determined the mature N-termini of hundreds of proteins from the model diatom *Thalassiosira pseudonana*, revealing extensive N-terminal modification by acetylation and proteolytic processing in both cytosol and plastid. We identified 63 mature N-termini of nucleus-encoded plastid proteins, deduced their complete transit peptide sequences, determined a consensus motif for their cleavage by the stromal processing peptidase, and found evidence for subsequent processing by a plastid methionine aminopeptidase. The cleavage motif differs from that of higher plants, but is shared with other eukaryotes with complex plastids.

## Introduction

**Figure 1:**
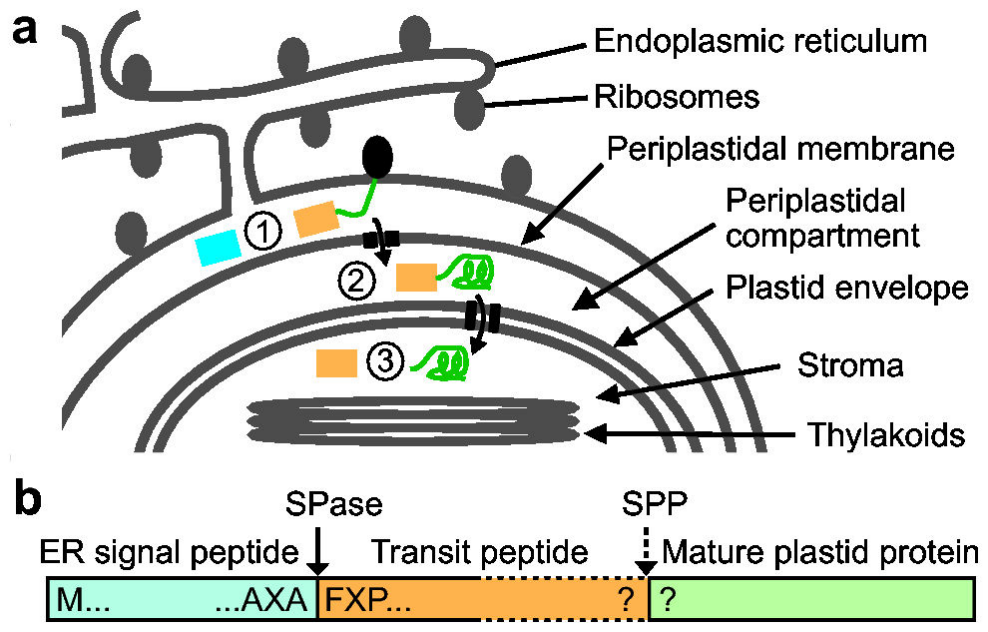
Protein import into complex plastids. (a) Schematic diatom plastid surrounded by four membranes, the outermost continuous with the ER. Proteins are synthesized on cytoplasmic ribosomes and co-translationally inserted into the ER lumen where the signal peptide (light blue) is removed by signal peptidase (1). The transit peptide (orange) then targets the proteins across the periplastidal membrane into the periplastidal compartment (2), and then through the translocons of the chloroplast double envelope into the plastid stroma, where the transit peptide is removed by the stromal processing peptidase (3). (b) Schematic structure of a nuclear-encoded plastid-targeted diatom protein precursor.

Algae with “secondary” or “complex” plastids derived from a red algal endosymbiont are widespread in all aquatic habitats, particularly the marine environment where they are a major part of the biota and significant contributors to global carbon drawdown [1]. These algae include the dominant diatoms, as well as brown seaweeds and other photosynthetic heterokonts (stramenopiles), haptophytes and cryptophytes. Their complex plastids originated via secondary endosymbiosis, where a non-photosynthetic eukaryote engulfed a red alga with a primary plastid, eventually retaining only the plastid and some red algal nuclear genes [2-4]. One consequence of this evolutionary process was that secondary plastids are surrounded by four rather than two bounding membranes (Figure 1a). The outermost membrane is part of the host’s endomembrane system, whereas the next innermost membrane, termed the periplastidal membrane, is believed to be derived from the red algal plasma membrane. The two innermost membranes originate from the endosymbiont’s outer and inner plastid envelope membrane.

In spite of the importance and global abundance of these algal groups—up to 25% of the photosynthetic capacity of plant earth—we are only beginning to understand how cellular transport systems evolved to allow nuclear-encoded proteins synthesized on cytoplasmic ribosomes to cross four membranes to arrive in the plastid stroma [5,6]. In all photosynthetic eukaryotes, the majority of plastid-located proteins are encoded in the nuclear genome, translated on cytoplasmic ribosomes and then transported into the plastid. In the case of primary plastids, a targeting sequence (transit peptide) directs their transport across the two plastid envelope membranes via the outer (TOC) and inner (TIC) translocation complexes [7,8]. For secondary plastids surrounded by four membranes, the chloroplast precursors require an N-terminal bipartite targeting sequence (Figure 1b) consisting of a typical endoplasmic reticulum (ER) signal sequence (SP) followed by a transit peptide sequence (TP) [9,10]. The TP of secondary plastids must therefore have three roles: targeting plastid proteins across the periplastidal membrane while preventing them from entering the secretory pathway, helping to maintain plastid proteins in an import-competent state in the periplastidal compartment, and engaging the TOC apparatus of the outer chloroplast envelope.

In an number of elegant studies using green fluorescent protein (GFP) fusions transformed into the diatom *Phaeodactylum* *tricornutum* , it was shown that the presence of a large hydrophobic residue (usually Phe but occasionally Tyr, Trp or Leu) immediately following the SP cleavage site is essential for correct targeting across the inner three membranes. This was generalized into the consensus motif ASA-FAP (Figure 1B) [9,10]. The N-terminal part of the TP is enriched in hydroxylated and depleted in acidic amino acids, resulting in a net positive charge. However, because the mature N-terminal sequences of only a handful of plastid proteins have been determined [11], there is little information about the C-terminal part of the TP or its cleavage site. To better understand protein import into secondary plastids, detailed information on the global properties of TPs, such as overall length, amino acid composition and the motif(s) for their cleavage in the plastid stroma, is required. Since TPs are degraded by the stromal processing peptidase (SPP) before being released [12], information about their C-termini can only be obtained by determining the mature N-terminal sequences of nuclear-encoded plastid-targeted proteins.

Several recent techniques for the high-throughput identification of protein N-termini have been developed [13]. Here we adapted the Terminal Amine Isotope Labeling of Substrate (TAILS) approach to identify native mature N-termini, whether or not they are naturally modified [14,15]. We identified mature N-terminal sequences of hundreds of proteins from the marine model diatom *Thalassiosira pseudonana* and studied their post-translational modification by N-terminal acetylation, N-terminal methionine excision and proteolytic processing. This collection included the N-termini of 63 plastid-targeted proteins, which allowed us to deduce the complete TP sequences of their precursors and to derive a generalized TP cleavage site motif.

## Materials and Methods

### Cell Lysis and protein preparation

Axenic cultures of *T. pseudonana* clone CCMP1335 were grown in enriched artificial seawater (ESAW) medium [16] at 18 °C, at 40 µmol photons m^-2^s^-1^ on a 12/12 photoperiod and harvested in exponential growth phase. Cells were pelleted (3000 g, 10 min, 4°C), washed twice with lysis buffer (50 mM HEPES, 20 mM KCl, 1 mM EDTA, 0.2 mM DTT, 150 mM sorbitol, pH 7.5) supplemented with PMSF and Complete protease inhibitor mixture (Roche), then broken with 0.3 mm glass beads in a mini-bead-beater (Omni International). The beads were removed by centrifugation (1,000 g, 5 min and the supernatant divided into crude soluble and membrane protein fractions by centrifugation at 16,000 g for 15 min. The supernatant fraction was filtered through a 0.22 µm filter, then concentrated and buffer exchanged to 50 mM HEPES (pH 7.5) using a spin filter device (3 kDa MW cutoff, Millipore). Aliquots of the membrane protein fraction were further purified by sucrose gradient centrifugation, diluted 10 times with 50 mM HEPES (pH 7.5) supplemented with complete protease inhibitor cocktail, sedimented at 45,000 g for 1 h and resuspended in 50 mM HEPES buffer pH 7.5 containing 1% deoxycholate. After heating for 1 min at 95 °C, the non-solubilized materials were pelleted at 14,000 g and the supernatants used for further analysis. All protein fractions were independently used for enrichment of N- terminal peptides.

### Enrichment of protein *N-*terminal peptides

**Figure 2:**
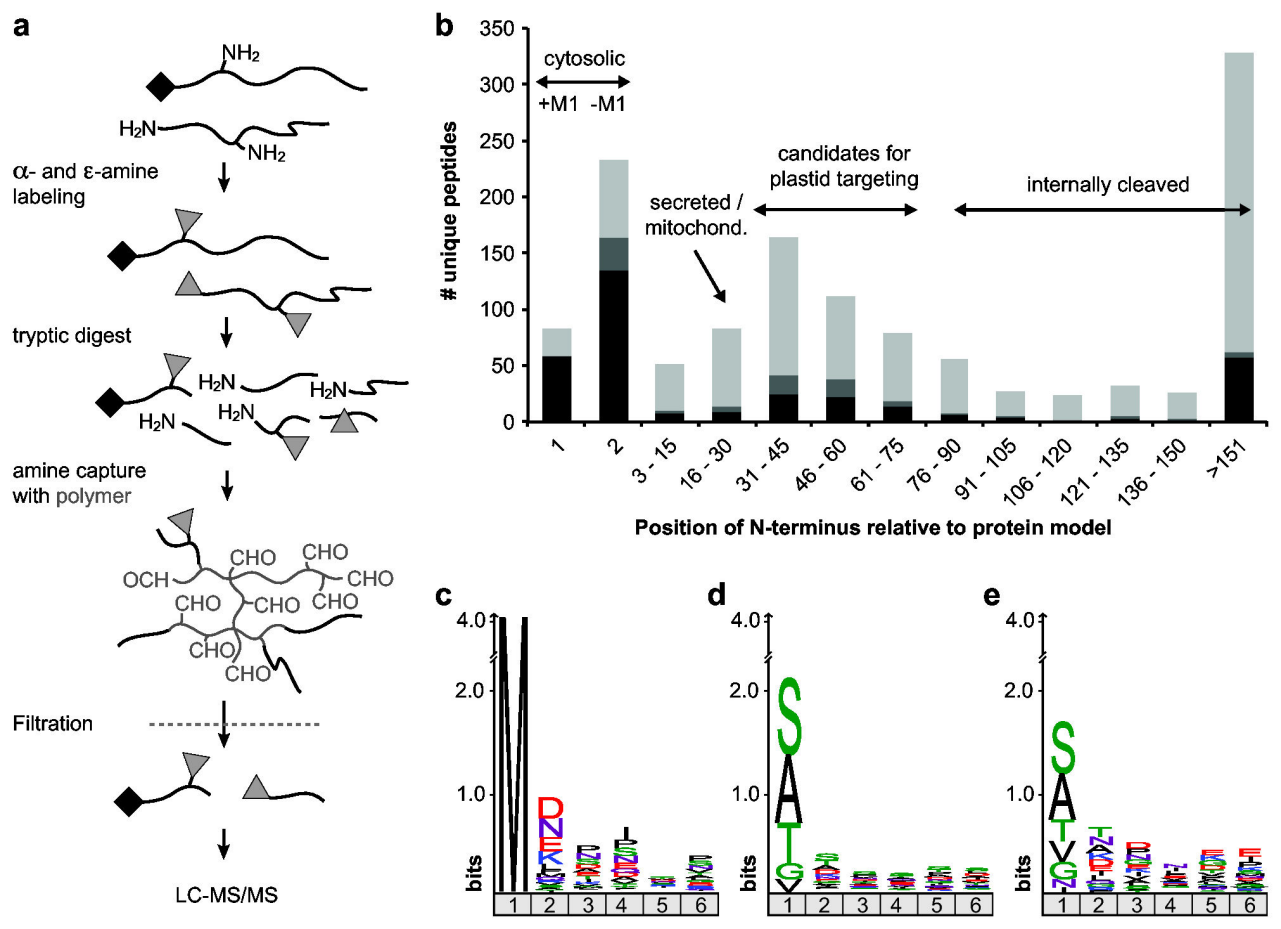
*T. pseudonana* protein termini identified by TAILS. (a) Schematic representation of the TAILS workflow. Proteins with free or naturally modified (black square) N termini are denatured, followed by chemical modification of all primary amines (grey triangle). Specific digestion with trypsin generates peptides amenable to mass spectrometric identification. N-terminal peptides are blocked, whereas internal or C-terminal peptides exhibit a trypsin-generated primary amine at their N terminus that is used to covalenty bind these peptides to an aldehyde-containing polymer which is subsequently removed by filtration. (b) Position of identified N-terminal peptides with respect to curated protein model. N termini matching the protein models at positions 1 and 2 are cytosolic proteins with intact (+M1) or removed (–M1) initiating Met. Black, acetylated N termini; dark grey, protein N termini present in both dimethylated and acetylated forms; light grey, free N termini identified as dimethylated peptides. (c) Sequence logoplot of the first 6 amino acids of 81 N termini of nuclear encoded proteins with intact initiating Met. (d) Sequence logoplot of 231 N termini of nuclear-encoded proteins starting at protein model position 2 because the initiating Met was removed. (e) Combined logoplot of N termini of 22 plastid-encoded proteins starting at position 2 after N-terminal Met excision plus 18 plastid-imported proteins with Met directly preceding the identified peptide.

Protein N-terminal peptides were enriched by a polymer-based negative selection method [17]. In short, 500 µg to 1 mg protein from each preparation were denatured, reduced, alkylated, followed by reductive dimethylation of the primary N-terminal α-amines and the ε-amines of Lys side chains using formaldehyde and sodium cyanoborohydrate (ALD coupling solution, Sterogene). Modified proteins were precipitated with chloroform/methanol, resuspended at 1 mg/ml and digested with 1 µg trypsin / 100 µg protein (Trypsin Gold, Promega). After digestion, the peptides with internal trypsin-generated primary amines were covalently bound to water-soluble high molecular weight dentritic polyglycerol-aldehyde polymer (HPG-ALD, Flintbox) using sodium cyanoborohydrate at a ratio of 2.5 mg polymer / mg peptides (Figure 2a). The polymer with coupled tryptic peptides was then removed from the unbound N-terminal peptides (which were either naturally amino-modified or chemically modified by the reductive dimethylation step) by filtration with a spin filter device (10 kDa MW cut-off, Millipore), desalted and identified by high resolution LC-MS/MS.

### LC-MS/MS

Enriched N-terminal peptides were desalted with C18 stop-and-go extraction tips or fractionated and desalted with C18-SCX-C18 stop-and-go extraction tips as described [18] prior to LC-MS/MS analysis with a linear ion trap–fourier transform ion cyclotron resonance mass spectrometer (LTQ-FTICR, Thermo) or linear ion trap-orbitrap mass spectrometer (LTQ-Orbitrap Velos, Thermo). The LTQ-FTICR and LTQ-Orbitrap instruments were coupled on-line to an Agilent 1100 Series and Agilent 1200 Series HPLC instrument, respectively, which separated peptide samples on columns packed with ReproSil Pur C18 beads using a 6–80% gradient of organic phase over 95 min. Buffer A was 0.5% acetic acid and buffer B was 0.5% acetic acid with 80% acetonitrile. The LTQ-FT was set to acquire a full-range scan at 25,000 resolution from 350 to 1,500 m/z in the FT-ICR cell, followed by selected ion monitoring (SIM) of the top three peptide ions in each cycle at resolution 50,000 for accurate mass measurement and MS/MS of these 3 ions in the LTQ (minimum intensity 500 counts). Parent ions were then excluded from MS/MS for the next 180 sec. The LTQ-Orbitrap was set for acquisition of full-range scans from 350 m/z to 1,500 m/z at a resolution of 60,000, followed by fragmentation of the five most intense ions in the LTQ. Raw data files were acquired by Xcalibur v2.0.1 (for LTQ-FTICR, Thermo) and Xcalibur v2.1.0 (for LTQ-Orbitrap Velos, Thermo).

The raw data associated with this manuscript may be downloaded from the ProteomeCommons.org Tranche network using the following hash:

0 eoIDZbMgFjoLK5DiSKJKDcYZcVzOFJNjxiT5l8nIrS3C32mygEYHb4Fx4xlgBeBEl244gJxMWyWe7OAtAHysPFZTFgAAAAAAAACsQ==

The hash may be used to prove exactly what files were published as part of this manuscript’s dataset, and the hash may also be used to check that the data has not changed since publication. The passcode for accessing the data is “ThapsTAILS”.

### Data analysis and identification of putative transit peptide sequences

For peptide identification, a *T. pseudonana* protein database was assembled combining the Joint Genome Institute (JGI) gene catalog protein models (downloaded from genome.jgi-psf.org/Thaps3/Thaps3.home.html on 2011-09-14) and the plastid-encoded protein sequences (downloaded from chloroplast.ocean.washington.edu on 2011-09-14) with appended reverse decoy sequences. For peptide identification with two search engines, X! TANDEM [19] and MASCOT v2.3 (Matrix Science), raw data files were converted to the mzXML format using ReAdW v4.3.1 (Institute for Systems Biology, Seattle) and mgf format using Proteome Discoverer 1.2 (Thermo Scientific). A custom Perl script completed the headers in the *. mgf files (added time and file name). Search parameters included: Precursor mass tolerance, 10 ppm; Fragment ion mass tolerance, 0.4 Da; enzyme, semi-ArgC (due to the inability of trypsin to cut at dimethylated Lys) with up to two missed cleavages, Peptide modifications: Carboxyamidomethylation of cysteine residues (+57.021464 Da), dimethylation of lysine ε-amines (+28.0313 Da) and variable N-terminal modification by acetylation (+42.010565 Da) or dimethylation (+28.0313 Da), Peptide identifications were subjected to secondary validation with PeptideProphet [20] as implemented in the Trans-Proteomic Pipeline v4.4 [21] and accepted with an estimated FDR <5%. Next, peptide lists from the database searches were combined into a non-redundant peptide list using an in-house script that also summed the number of spectra supporting each unique identified sequence across different experiments. Peptides with unmodified N termini carried over from incomplete depletion were removed from the results list, as were peptides not ending with Arg. The start position of each peptide in relation to the protein model was calculated, and protein annotation information retrieved from Uniprot, JGI gene catalog and CpBase supplementary data as available.

The position of the identified N-terminal peptide in relation to the protein sequence derived from the gene model was used for pre-selection of candidate plastid proteins. This was followed by manual examination of all gene models for which identified N-terminal peptides matched between residue positions 16 and 75, unless the available annotation information indicated a non-plastid destination for the protein. Classification as the mature N terminus of a plastid-targeted protein required an N-terminal ER SP sequence, followed by Phe or Tyr in a variation of the known FXP or FXXP motif [9,10]. In a number of cases, the sequences coding for the ER signal peptides required for the first step of plastid import were found only after upstream extension of incomplete gene models.

To find homologs in other algae with red plastids, the draft genomes available at JGI (http://genome.jgi-psf.org/) were searched using AlgaeBlast (http://genome.jgi-psf.org/Algae/Algae.info.html). Related sequences were also obtained from Genbank (http://blast.ncbi.nlm.nih.gov/).

Sequence logos for aligned sequences were generated using the iceLogo webserver [22].

## Results

### Identification and characterization of protein *N-*terminal peptides

Diatom cells are surrounded by a rigid silicaceous wall (frustule), which can only be broken by agitation with glass beads or even more drastic measures that also disrupt the fragile plastids. For this reason, total *T. pseudonana* lysates fractionated into crude soluble and membrane fractions by centrifugation had to be used. These fractions were independently enriched for N-terminal tryptic peptides using TAILS (Figure 2a) [14]. In TAILS, free N-terminal α-amino groups of proteins that are not modified in vivo, e.g. by acetylation, and lysine ε-amino groups are first blocked in whole proteins by reductive dimethylation. Thus, after tryptic digestion, all peptides originating from true protein N termini are either chemically or naturally blocked, whereas trypsin-generated peptides display primary α-amines that are then covalently coupled to a water-soluble aldehyde-functionalized polymer. The high-molecular weight polymer with bound internal peptides is removed by spin filtration, leaving a filtrate highly enriched in N-terminal peptides.

Enriched N-terminal peptides were analyzed by high resolution LC-MS/MS and individual peptide sequences were identified from a database containing all *T. pseudonana* protein models predicted from the nuclear and plastid genomes using two different search engines (see Methods). Spectrum-to-sequence assignment searches considered only dimethylation or acetylation as N-terminal modifications, since most other naturally occurring N-termini blocking modifications are rare and affect only a low percentage of proteins [23]. The resulting peptide assignment lists from different experiments and searches (Tables S1 to S4) were combined into a non-redundant peptide list for further analysis.

We identified a total of 1,401 distinct N-terminal peptide sequences. Of these, 1,055 peptides were dimethylated, i.e. originating from proteins that had N-termini with free α-amines in vivo, and 438 were acetylated, i.e. originating from proteins with co- or post-translationally acetylated N-termini. In addition, 92 N-terminal peptides were found in both dimethylated and acetylated forms, indicating partial acetylation in vivo. 1,295 of the peptides matched 939 proteins encoded by the nuclear genome (Table S5), and 106 peptides matched 37 chloroplast-encoded proteins (Table S6).

The N-terminal peptides were sorted into bins according to where they mapped on the matching protein model sequence (Figure 2b). N-termini of nuclear-encoded proteins starting at the first or second amino acid of the model were assumed to be primarily cytosolic, i.e. polypeptides without any organellar or secretory targeting presequence. Proteins with mitochondrial or ER signal sequences should fall in the 16 to 30 residue bin, while a small number of N-termini in bin 3-15 result from limited processing, mostly by amino-peptidases. The best candidates for proteins with a bipartite plastid-targeting sequence were expected in the 30-75 residue bins. Peptides mapping more than 75 amino acids from the beginning of the protein model were regarded as products of internal endoproteolytic processing and were not examined further. However, it should be noted these “internal” N termini result from common physiological processes as well as from technical limitations: i) splicing and alternative translation starts; ii) proteolytic processing, a post-translational modification that regulates the function of many proteins, e.g. zymogen activation [15]; iii) unusually long targeting sequences, e.g. in proteins targeted to the thylakoid lumen; iv) naturally occurring degradation intermediates of abundant proteins that, despite their short half-lives, can be present in higher concentrations than low-abundance proteins; v) background proteolysis during sample preparation by proteases resistant to the inhibitor cocktail used; vi) incorrectly predicted protein models. The proportion of endoproteolytic products (38% mapping at positions >75) may appear surprisingly large, but is comparable to the proportion of “internal” N termini observed in other studies, e.g. mouse tissues (44% [24]) and human Jurkat cell lysates (51% [25]) and thus likely reflects the proportion of proteolytic processing and accumulation of degradation intermediates encountered naturally in vivo.

Almost three-quarters of the cytosolic proteins identified at their predicted protein start had their initiating Met removed (bin 2) as part of their co-translational processing in the cell [23]. In agreement with general specificity rules for Met aminopeptidases [26], the initiating Met was retained if the penultimate residue was a charged residue, e.g. Asp, Asn or Glu (Figure 2c), and removed if the penultimate residue had a small gyration radius, e.g. Ala, Gly, Ser, Thr, or Val (Figure 2d). This shows that diatom Met aminopeptidases follow the same rules as those of other eukaryotes [23,27]. Complete or partial N-terminal acetylation was observed for 70% of the cytosolic proteins, irrespective whether the initiating Met was retained (Figure 2B, bin 1) or removed (Figure 2B, bin 2). Thus N-terminal acetylation is more frequent than reported for yeast (60%), in agreement with increasing percentages of N-terminal acetylation towards more complex eukaryotes [28].

### Identification of plastid-targeted proteins

Proteins with N-terminal peptides matching between position 30 and 75 of the corresponding gene model and those with annotations suggesting a plastid location were considered candidates for chloroplast-targeted proteins. Each of the protein models was manually inspected unless the available annotation information clearly indicated a non-plastid destination. Classification as a plastid-targeted protein required that the model had a potential N-terminal bipartite targeting sequence, i.e. an ER signal peptide sequence followed by F or Y (Figure 1b). In a number of cases, the ER signal peptide sequence required for the first step of plastid import was only found after re-evaluation and upstream extension of the *T. pseudonana* gene models. In total, more than 500 sequences were subjected to expert manual evaluation.

**Figure 3:**
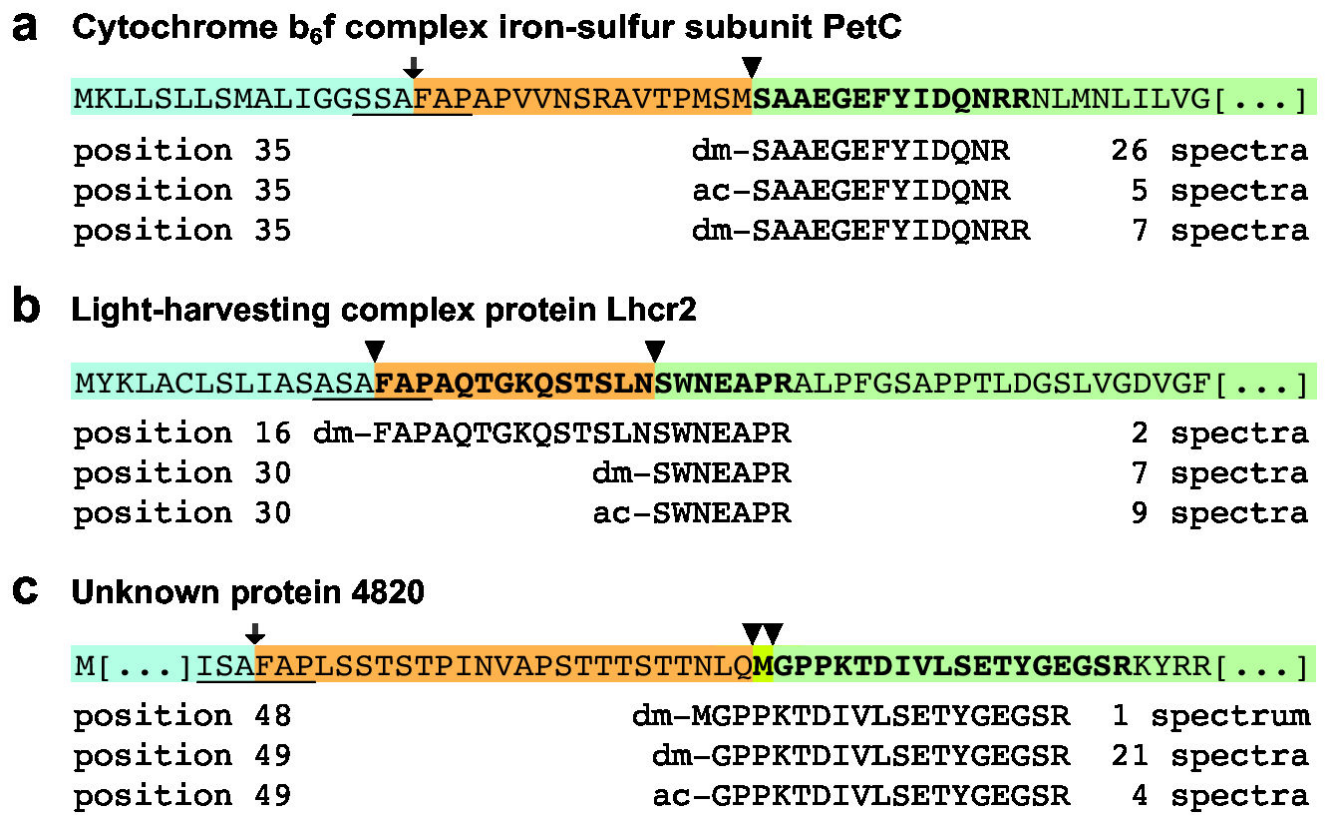
Approach for determining diatom transit peptide sequences. Transit peptides (orange) and transit peptide cleavage sites were deduced by mapping identified N termini (green) to the protein model sequence after removal of the ER signal sequence (blue). (a) Cytochrome b_6_f complex iron-sulfur protein subunit (PetC). Three peptides identify a single unique N terminus at position 35 of the protein model. (b) Light harvesting antenna complex protein Lhcr2. An acetylated and a dimethylated peptide identify the mature protein N terminus at protein model position 30, and a dimethylated peptide begins at the canonical SP cleavage site (ASA-FAP) at protein model position 16, indicating that this protein was incompletely processed or in transit when isolated. (c) Unknown protein 4820, homologous to a putative higher plant plastid precursor protein. Two peptides identify a mature, partially acetylated N terminus starting at protein model position 49, while a third peptide has an N-terminal Met starting at position 48. Bold, observed peptides; underlined, conserved ASA-FAP motif; arrow, inferred ER signal peptide cleavage site; arrowhead, observed protein termini.

As an example, Figure 3a shows the N-terminal peptides derived from PetC, the Rieske iron-sulfur protein of the plastid cytochrome b_6_f complex, which is known to be nuclear-encoded and translated on cytoplasmic ribosomes. Two unique peptide sequences, differing by a missed tryptic cleavage site at their C termini, suggest a single unique N terminus for PetC starting at Ser-35. One peptide was found in both acetylated and dimethylated forms, with approximately one-seventh of the spectra corresponding to the acetylated form. Since acetylation occurs only in vivo, this further supports the identification as the true N terminus of mature PetC. When matched to the complete precursor sequence, the sequence between the SP cleavage site and the mature N terminus suggests a TP sequence 17 amino acids in length. A second example is the Chl *a/c* light-harvesting protein Lhcr2 (Figure 3b), where two different N-terminal peptides were identified. The first started with FAP, indicating that the protein still had an intact TP at the time of sampling. In addition, 16 spectra defined a unique partially acetylated mature N terminus at Ser-35, from which a 14-residue TP sequence was deduced. A third example is the unknown protein 4820, which is homologous to a putative plastid precursor protein in vascular plants (Figure 3c). Here, two well-supported peptides define a unique, partially acetylated mature N terminus at Gly-49 of the protein model, from which a 26-residue TP is deduced. However, an additional dimethylated peptide indicates an alternative start at Met-48.

This approach led to the identification of 63 precursor proteins with a predicted SP cleavage site followed by Phe or Tyr, and an experimentally determined mature N terminus 13 to 42 amino acids further downstream (Table S7). The proteins were mostly those typical of a plant or green algal chloroplast proteome [29,30]. They included 14 members of the Chl *a/c* (LHC) protein family, 7 enzymes of heme or Chl biosynthesis, various enzymes of glycolysis, Calvin-Benson cycle, amino acid biosynthesis, carotenoid biosynthesis, lipid biosynthesis and sulfate reduction, 2 elongation factors (EF-Ts, EF-G), the protease subunit ClpB and a putative bicarbonate transporter (Table S7). The list also included a putative plastid-targeted N-acetyl transferase, which may be responsible for the observed N-terminal acetylation of imported and plastid-encoded proteins. Finally, we identified 14 proteins with unknown function that contained a SP followed by a canonical FAP or FXXP. Of these, 12 had homologs in at least one other diatom in the JGI algae databases.

### Identification of the transit peptide cleavage motif and subsequent Met removal

**Figure 4:**
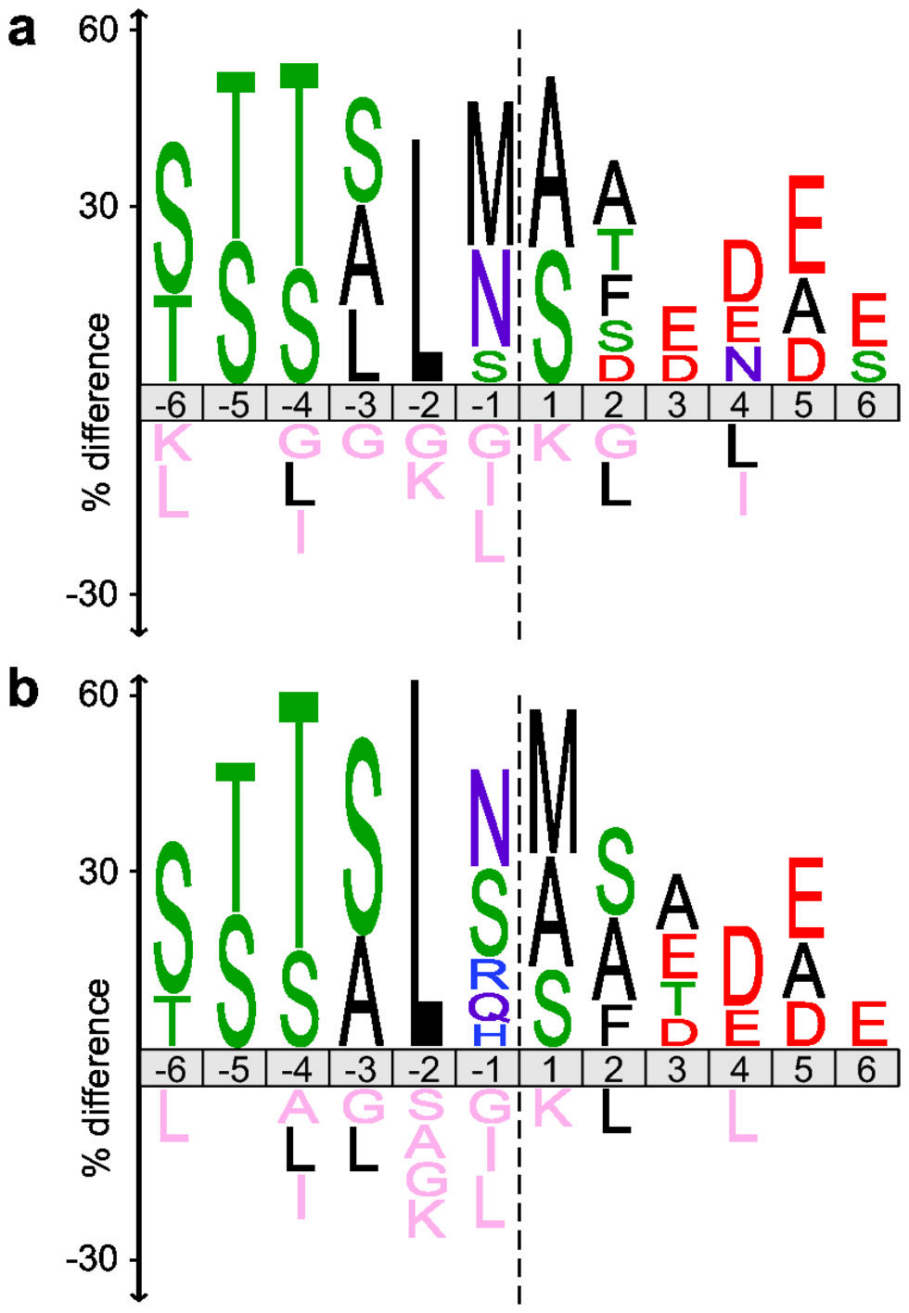
Amino acid occurrences at the transit peptide cleavage site. IceLogos of sequences surrounding the putative transit peptide cleavage site (dotted line) of 63 plastid-imported proteins (a) based on alignment of the identified N-terminal sequences and the C-terminus of the deduced transit peptides. (b) based on an alignment of the same 63 sequences, with the 18 sequences that showed a Met at -1 shifted by one position to the right on the assumption that N-terminal Met was removed by a plastid Met-aminopeptidase after import and transit peptide cleavage. Note that iceLogos show the difference between the observed amino acid frequency among the 63 identified sequences and the natural amino acid abundance in *T. pseudonana* proteins, i.e. overrepresented amino acids are indicated above the line and underrepresented amino acids below the line. Only differences with a p-value of 0.05 or smaller are shown.

The residues bracketing the deduced TP cleavage sites were plotted as an iceLogo (Figure 4a), which shows residues that are significantly over- or underrepresented at each position compared to the natural abundance of each amino acid in the *T. pseudonana* proteome [22]. The strongest preference for any particular amino acid was not directly at the cleavage site but was a Leu at either the -2 or -3 position. The occurrence of Leu at the -3 position strongly correlated with Met at the -1 position, whereas none of the 63 identified N-terminal peptide sequences started with Met. Since removal of N-terminal Met is ubiquitous in both prokaryotes and eukaryotes [23,26], we hypothesized that in these cases the initial TP cleavage occurred just before a Met that was subsequently removed by a Met-specific aminopeptidase residing in the plastid.

Our data for plastid-encoded proteins synthesized on plastid ribosomes supported this interpretation. We identified N-terminal peptides from 37 out of the 118 plastid-encoded proteins [31], all of which would have been synthesized starting with Met (Table S6). Of those peptides mapping close to the beginning of the gene model, 5 retained their N-terminal Met while 22 started with the second amino acid. A sequence logo plot based on these 22 N termini from Met-processed plastid-synthesized proteins plus the 18 N-terminal peptides with a preceding Met from imported proteins showed that the next amino acid after an excised Met was usually one favored by Met aminopeptidases [26], i.e. Ser, Ala, Val, Thr and Gly (Figure 2e).

When the iceLogo for the TP cleavage site was replotted with the assumption that N-terminal Met were removed after TP cleavage by the stromal processing peptidase (SPP), there was a clear, very strong preference for Leu at position -2 (Figure 4b). In addition, there was a strong preference for hydroxylated residues at positions -3 to -6. However, a wider range of amino acids was observed just before the SPP cleavage site (position -1). There appeared to be a weak preference for small or amidated amino acids, while large aliphatic residues were not found. On the other side of the cleavage site, the first residue after the cleavage site was most frequently Met, Ala or Ser, and the next 5-8 residues of the mature proteins were enriched in negatively charged side-chains.

The final deduced TP sequences ranged from 12 to 42 amino acids in length with an average of 20 (Table S7). Like plant TPs [7], they were highly enriched in Ser and Thr residues (18% and 14%, respectively) and almost completely depleted in acidic residues (Asp and Glu, 0.3% and 0.6%, respectively), resulting in a net positive charge in all but two TPs, with an average of +2.6. The positively charged residues (Arg, Lys) tended to be in the middle of the TP, but the hydroxylated residues were found throughout. Leu and Ile are notably absent or under-represented in positions -1 and -3 to -6 (Figure 4b).

### Generality of the transit peptide cleavage motif

**Figure 5:**
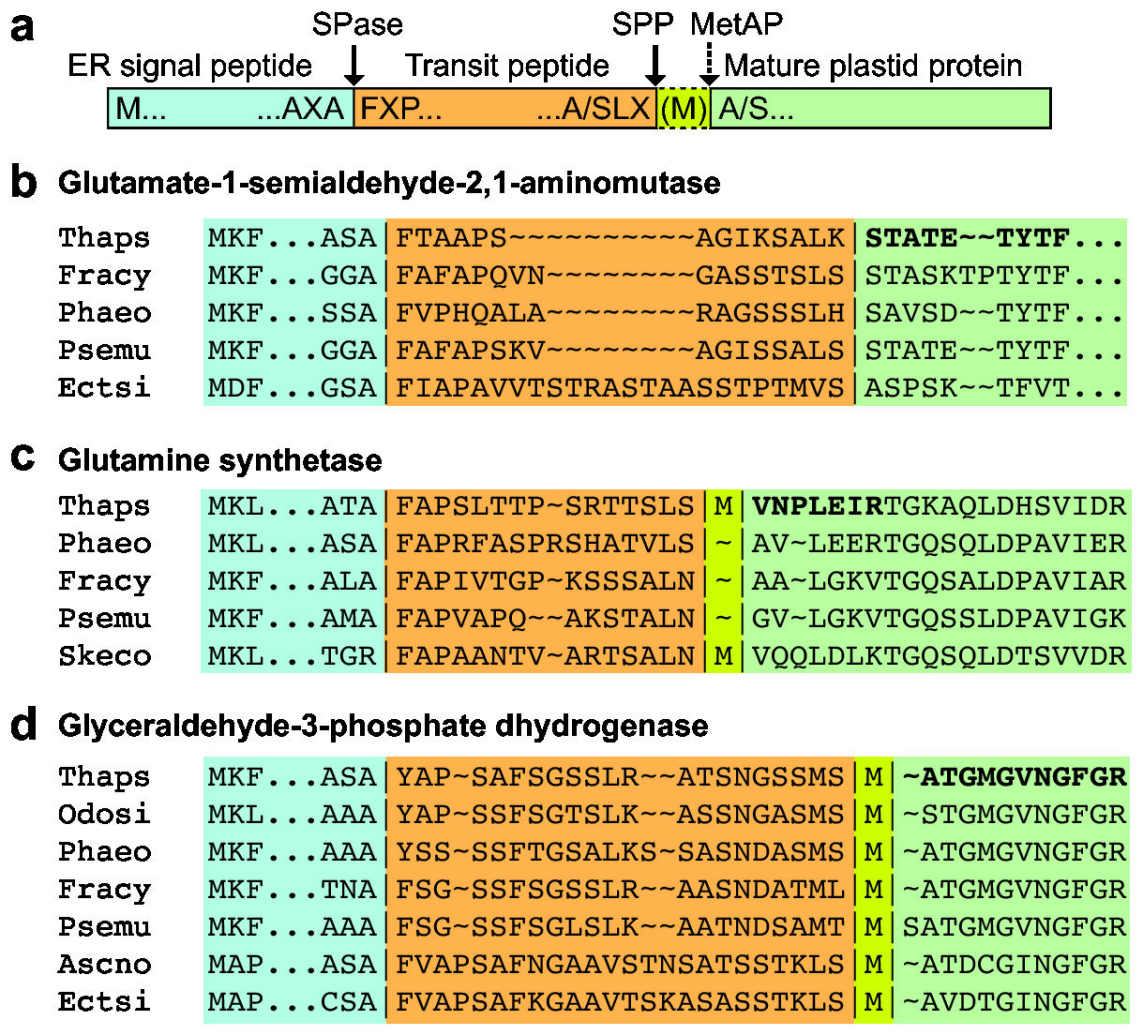
Conserved processing sites in related proteins from other organisms with complex plastids. Processing sites for related proteins of other heterokonts were predicted based on alignments of mature protein sequences. (a) Schematic structure of a nuclear encoded plastid-targeted diatom protein. Proteolytic processing steps and proposed cleavage site consensus sequences are indicated. (b) Alignment of glutamate-1-semialdehyde 2,1-aminomutase from four diatom species and a brown alga (c) Alignment of glutamine synthetase from three diatom and two brown algal species. (d) Alignment of glyceraldehyde-3-phosphate dehydrogenase from five diatoms and two brown algae. Bold letters, mature *T. pseudonana* N terminal sequences identified in this study. Light blue, SP; orange, TP; yellow, a Met that may be removed from the N terminus after SPP processing; green, mature plastid stroma-targeted protein. Thaps, *Thalassiosira pseudonana*; Phaeo, *Phaeodactylum* *tricornutum* ; Fracy, *Fragilariopsiscylindrus* ; Psemu, *Pseudo-nitzschia* *multiseries*; Skeco, *Skeletonema* *costatum* ; Odosi, *Odontella* *sinensis* ; Ascno, *Ascophyllum* *nodosum* ; Ectsi, *Ectocarpussiliculosis* .

Our data suggest that diatom TPs are cleaved at a consensus site with Ala or Ser at -3, Leu at -2, and a charged or small amino acid at -1. The mature N terminus generated by cleavage is usually Ala, Ser or a subsequently removed Met and the next 5-6 amino acids have a negative net charge. In order to examine whether these "rules" for TP cleavage sites are similar in other algae with secondary plastids, databases were searched for homologous gene models encoding a SP followed by the canonical (F/Y) XXP motif. Almost all the proteins in our study (including unknown proteins) had a homolog in at least one other diatom, and many had homologs in other algae. Unfortunately, gene models are often incomplete at the 5'-end or have introns in the targeting sequence, so cannot be aligned in the region of the predicted cleavage site and mature N-terminus. Three representative examples of proteins where it was possible to examine the cleavage site motif across several species are shown in Figure 5.

**Figure 6:**
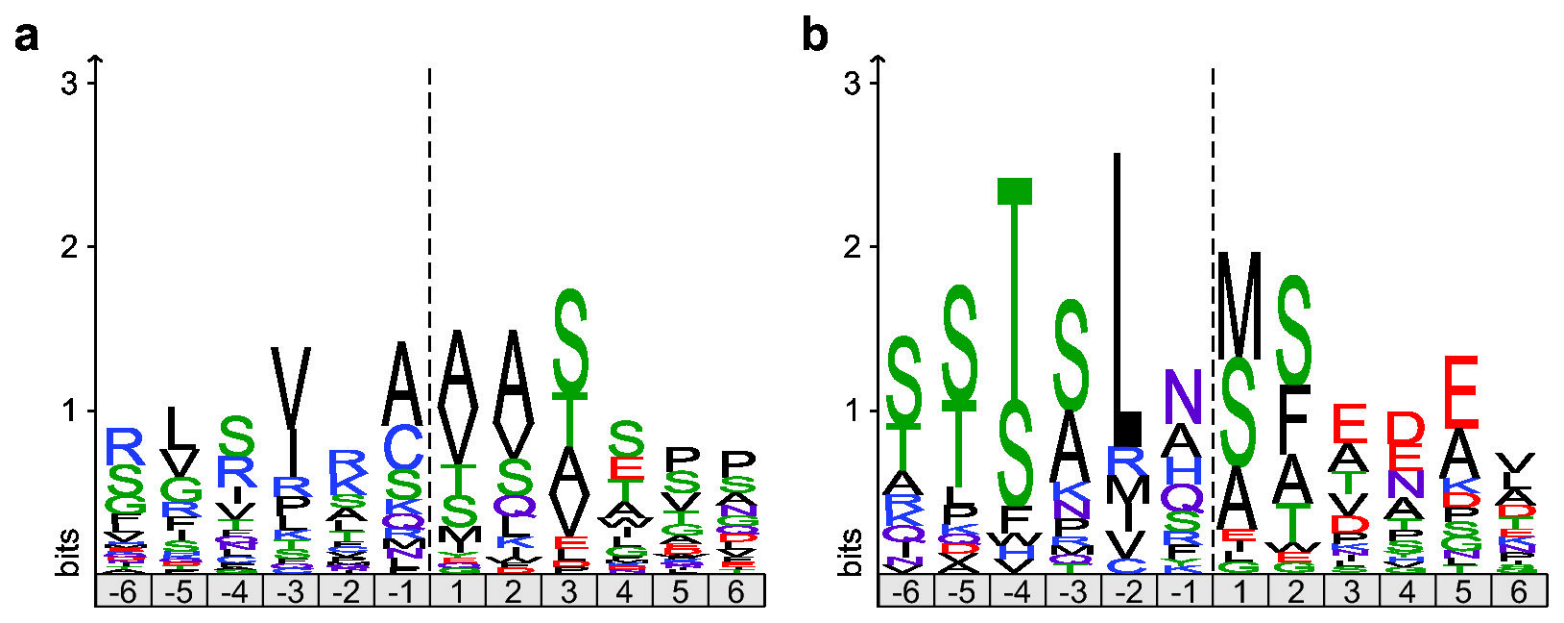
Comparison of transit peptide cleavage sites in higher plants and diatoms. (a) Sequence logo based on 47 acetylated N-termini of chloroplast imported *A. thaliana* proteins identified by Zybailov et al. as semi-tryptic peptides within 10 amino acids from the predicted cleavage site [29]. (b) Sequence logo based on 30 acetylated N-termini of plastid-targeted *T. pseudonana* proteins identified in this study. Only acetylated N-termini were used to exclude a potential bias from comparing acetylated sequences from *A. thaliana* with non-acetylated ones from *T. pseudonana*.

For glutamate-1-semialdehyde 2,1-aminomutase, the sequences from three additional diatoms and the brown alga *Ectocarpussiliculosis* (Ectsi) aligned very well with the identified *T. pseudonana* N terminus. All four diatom TPs contained a Leu at the -2 position of the predicted TP, while the *E* *. siliculosis* TP has Val at this position (Figure 5B). For glutamine synthetase, alignment of five diatom sequences predicted that two of the five (*T. pseudonana* and *Skeletonema* *costatum* ) would have an Met preceding the mature N terminus. If subsequent plastid Met processing after TP cleavage is taken into account, all five sequences would have a Leu at the -2 position, preceded by several hydroxylated residues (Figure 5C). In the case of GAPDH, the mature N termini of five diatoms and two brown algae ( *E* *. siliculosis* and *Ascophyllum* *nodosum* ) would all be preceded by a Met. If Met processing after TP cleavage is taken into account, all five diatoms would have another Met at the -2 position of the inferred TP cleavage site and lack a negative net charge at the protein N terminus, while the two aligned brown algal sequences would have the canonical Leu at -2 and a net negative charge in the following 6 amino acids (Figure 5C). It therefore appears that there is a conserved transit peptide cleavage site motif, at least for diatoms and brown algae.

## Discussion

Protein import across the four membranes surrounding a secondary plastid is much more complex than import into the primary plastids of plants. The only known import pathway requires a bipartite targeting sequence composed of a canonical ER SP followed by a TP that directs the precursor protein across the inner three membranes [6]. The first import step is well-understood, but lack of experimental information about the TP has impeded the study of the subsequent steps. Since targeting sequences are removed and degraded before proteins reach their final destination, their sequences and cleavage sites must be determined indirectly. In this study we selectively enriched and identified N-terminal peptides of 977 nuclear- and plastid-encoded proteins from the diatom *T. pseudonana*. Among these, we were able to determine mature N-terminal sequences of 63 nuclear-encoded proteins imported into the plastid stroma using the presence of a clearly discernable bipartite targeting sequence with canonical ER SP cleavage site as a strict selection criterion. From this we could deduce the sequences of the second part of the bipartite targeting sequences and determine the general properties of diatom TPs, including the consensus sequence for processing by SPP. However, we wish to emphasize that our approach does not capture any proteins that might use atypical SP cleavage sites or be targeted via as yet undiscovered alternate pathways, similar to those described for primary plastid import in higher plants [8]. As there were few clearly annotated lumen-targeted proteins in our dataset, we did not further analyze their tripartite sequences.

Endogenous proteolytic processing complicated our analysis by generating multiple N termini for several proteins. However, identification of naturally acetylated N-terminal peptides for almost half of these proteins confirmed they were indeed true N termini, acetylated in the plastid after import and processing. Co-translational acetylation is a very common modification that affects between 50 and 80% of all eukaryotic proteins across all studied species [15,28,32]. Post-translational acetylation affects approximately 30% of stromal proteins in *Chlamydomonas reinhardtii* chloroplasts [33] and has also been described in higher plant plastids [29]. Both cytosolic and plastid proteins in *T. pseudonana* are no exception, and acetylated N termini showed similar sequence patterns as in other species (Figure S1) [27,32,34]. Notably, one of the proteins identified in our study was a plastid-targeted N-acetyl transferase, which may be one of the enzymes responsible for acetylation in the plastid.

The 63 deduced diatom TP sequences vary in length from 12 to 42 residues, i.e. they are shorter than plant TPs, which range from 20 to more than 100 residues [8]. Experimental studies with the N termini of diatom TPs fused to GFP have suggested that even shorter sequences could enable plastid import [10,35]. Diatom TPs may not need to be as long as plant TPs because they are hidden until the SP is cleaved off after the protein is already partly inserted into the ER, avoiding the danger of misdirection to the mitochondrion. The previously reported net positive charge, which was confirmed for almost all TPs identified in our study, is thought to be required for transit across the plastid envelope, but not for traversing the periplastidal membrane [36]. The TPs were enriched in hydroxylated amino acids that may play a role in keeping the precursor in an import competent state in the periplasm, possibly by interaction with resident chaperones [37,38]. It should be pointed out that the mature N termini could also contain targeting information [10,36,39]. Hence the negative charges that are usually found at the N termini of the mature diatom proteins may also contribute to targeting.

Once in the stroma, TPs are removed from the precursor by an endopeptidase (SPP). The diatom TP cleavage sites that we deduced here showed considerable variation in sequence, but in many cases had a Leu or other hydrophobic residue at -2 or -3. We noted that none of the identified mature N-termini started with a Met, but in 18 cases the genome-encoded protein sequence showed a Met just prior to the apparent cleavage site and this was strongly correlated with Leu at -3. We suggest that these Met were removed by a plastid-located Met aminopeptidase after SPP cleavage, supported by i) the observation of an example of incomplete N-terminal Met processing (Figure 3c), ii) the good agreement of the observed N-terminal amino acids with the known Met aminopeptidase specificity (Figure 2) [23] and iii) earlier reports of subsequent N-terminal processing of a subset of imported proteins in yeast mitochondria [40] and plant chloroplasts [41]. Furthermore, the resulting N-terminal amino acids were predominantly those that benefit protein stability according to the N-end rule [23,42,43], whereas retention of an N-terminal Met in the plastid might act as a destabilizing factor [44]. Taking N-terminal Met removal as a secondary processing step into account, we were able to infer a generalized consensus sequence for the hitherto elusive SPP cleavage site for diatom TPs.

We note that the *T. pseudonana* genome encodes a putative SPP homolog of the M16 metalloprotease family with an N-terminal bipartite targeting sequence, and that SPP activity has been detected biochemically in another heterokont, the raphidophyte *Heterosigmaakashiwo* [45]. Plant SPP recognizes imported proteins by binding to the C terminus of the TP sequence, an interaction that is strong enough to retain the TP bound to the enzyme after cleavage and release of the mature protein [12]. Intuitively, such a mechanism would be consistent with more restricted cleavage site specificity just upstream of the cleavage site, such as the strong preference for Leu at -2. A similar preference has been deduced for plant SPP, where branched hydrophobic are predicted at -2 [41], and demonstrated for the related mitochondrial processing peptidase, which showed a strong preference for Arg at -2 [40].

The diatoms and brown algae are prominent members of the Heterokonta and currently have the best quality genomic data of the species with secondary red plastids. Examination of homologs of the 63 *T. pseudonana* proteins suggests that the major features of the transit peptides and their cleavage site are conserved in the other heterokonts. But how do these TPs compare with the TPs of precursors targeted to primary plastids? Unfortunately, there is no proteomics data available for red algal plastid precursors. Most of the experimental studies on transit peptide cleavage have been done on higher plant proteins [29]. To compare the *Arabidopsis thaliana* and *T. pseudonana* cleavage site motifs, we generated sequence logo plots based on the 47 N-terminally acetylated semi-tryptic peptides of *A. thaliana* plastid proteins shown in Figure 6a of Zybailov et al. [29] and the 30 acetylated peptides of *T. pseudonana* identified in this study (Figure 6). Since acetylation only occurs vivo [28,29], these are the most rigorously identified mature plastid N-termini. In the case of the plant precursors (Figure 6a), there is a modest preference for the branched hydrophobic residues Val or Ile at -3, and for a small side-chain at -1. If additional trimming by plastidal aminopeptidases is considered, as suggested by Emanuelsson et al. [41], this correlates well with the strong preferences for Leu at -2 and a small, uncharged residue at +1 observed in *T. pseudonana* (Figure 6b). However, hydroxylated amino acids are not clustered between -6 and -3 in plant targeting sequences, and there is no indication of a preference for negatively charged residues in the first 6 positions of the mature protein. This suggests that different modifying factors may be involved in transit peptide cleavage and subsequent processing steps in primary and secondary plastids.

## Conclusions

Our targeted analysis of N-terminal sequences from the diatom *T. pseudonana* has provided the first insight into N-terminal modifications of diatom proteins by Met removal, N-terminal acetylation and the extent of proteolytic processing. This enabled us to deduce the complete TP sequences for 63 nuclear-encoded, plastid-imported proteins of the diatom *T. pseudonana*. We identified conserved sequence determinants for protein maturation by SPP and a stromal Met aminopeptidase after heterokont plastid import. With this information, we now have a comprehensive picture of the entire bipartite targeting sequence, including the properties of the transit peptide that allow plastid precursor proteins to traverse the periplastidal membrane, remain import competent in the periplasm, and engage the inner and outer translocons of the plastid envelope before maturation in the plastid stroma.

## Supporting Information

Figure S1 **N-terminal acetylation in *T. pseudonana*.** Sequence logos show the N-terminal 6 residues of (a) 58 acetylated N termini of nuclear-encoded proteins with intact Met at protein model position 1, (b) 164 acetylated N termini of nuclear-encoded proteins starting at position 2 after Met processing, (c) 206 acetylated N termini mapping to positions within the protein model, (d) 8 acetylated N-termini of plastid-encoded proteins starting at protein model position 2 after Met removal and (e) 30 acetylated N termini of nuclear-encoded, plastid-imported proteins.
(EPS)

Table S1 **List of 413 LTQ-FTICR spectra assigned to 311 peptides using Mascot.** MS/MS spectra aqcuired with a LTQ-FTICR mass spectrometer were searched against a *T. pseudonana* protein database using Mascot and evaluate with PeptideProphet as implemented in the TPP (for details, see methods section). Only peptide assignments with an estimated FDR <0.05 are listed. #, spectrum number; peptide, peptide sequence in standard one letter code; prob, PeptideProphet probability; ionscore, Mascot ionscore; z, charge; prec neutral mass, calculated precursor neutral mass; error [ppm], deviation of experimental peptide mass in ppm. Non-standard abbreviations in the peptide column: n[29.04], dimethylated peptide alpha-amine; n[43.02], acetylated peptide alpha-amine; C[160.03], carbamidomethylated cysteine; K[156.13], dimethylated Lys epsilon-amine; M[147.04], oxidized methionine.
(XLS)

Table S2 **List of 370 LTQ-FTICR spectra assigned to 269 peptides using X!** **Tandem**. MS/MS spectra acquired with a LTQ-FTICR mass spectrometer were searched against a *T. pseudonana* protein database using X! Tandem and evaluated with PeptideProphet as implemented in the TPP (for details, see methods section). Only peptide assignments with an estimated FDR <0.05 are listed. #, spectrum number; peptide, peptide sequence in standard one letter code; prob, PeptideProphet probability; hyperscore, X! Tandem hyperscore; z, charge; prec neutral mass, calculated precursor neutral mass; error [ppm], deviation of experimental peptide mass in ppm. Non-standard abbreviations in the peptide column: n[29.04], dimethylated peptide alpha-amine; n[43.02], acetylated peptide alpha-amine; C[160.03], carbamidomethylated cysteine; K[156.13], dimethylated Lys epsilon-amine; M[147.04], oxidized methionine.
(XLS)

Table S3 **List of 3392 LTQ-Orbitrap spectra assigned to 1848 peptides using Mascot.** MS/MS spectra acquired with a LTQ-Orbitrap Velos mass spectrometer were searched against a *T. pseudonana* protein database using Mascot and evaluated with PeptideProphet as implemented in the TPP (for details, see methods section). Only peptide assignments with an estimated FDR <0.05 are listed. #, spectrum number; peptide, peptide sequence in standard one letter code; prob, PeptideProphet probability; ionscore, Mascot ionscore; z, charge; prec neutral mass, calculated precursor neutral mass; error [ppm], deviation of experimental peptide mass in ppm. Non-standard abbreviations in the peptide column: n[29.04], dimethylated peptide alpha-amine; n[43.02], acetylated peptide alpha-amine; C[160.03], carbamidomethylated cysteine; K[156.13], dimethylated Lys epsilon-amine; M[147.04], oxidized methionine.
(XLS)

Table S4 **List of 3136 LTQ-Orbitrap spectra assigned to 1540 peptides using X!** **Tandem**. MS/MS spectra acquired with a LTQ-Orbitrap Velos mass spectrometer were searched against a *T. pseudonana* protein database using X! Tandem and evaluated with PeptideProphet as implemented in the TPP (for details, see methods section). Only peptide assignments with an estimated FDR <0.05 are listed. #, spectrum number; peptide, peptide sequence in standard one letter code; prob, PeptideProphet probability; hyperscore, X! Tandem hyperscore; z, charge; prec neutral mass, calculated precursor neutral mass; error [ppm], deviation of experimental peptide mass in ppm. Non-standard abbreviations in the peptide column: n[29.04], dimethylated peptide alpha-amine; n[43.02], acetylated peptide alpha-amine; C[160.03], carbamidomethylated cysteine; K[156.13], dimethylated Lys epsilon-amine; M[147.04], oxidized methionine.
(XLS)

Table S5 **List of 1295 unique N terminal peptides from 939 nuclear-encoded *T. pseudonana* proteins.** A non-redundant list of unique N terminal peptides, identified by database searches with Mascot and X! Tandem, was generated using an in-house script (see methods section for details). JGI ID, JGI protein model accession number; peptide, peptide sequence; position, peptide start position as matched to protein sequence; spectra (dimethylated), number of spectra identifying the N-terminally dimethylated form of this peptide; spectra (acetylated), number of spectra identifying the N-terminally acetylated form of this peptide.
(XLS)

Table S6 **List of 106 unique N terminal peptides from 37 plastid-encoded *T. pseudonana* proteins.** A non-redundant list of unique N terminal peptides, identified by database searches with Mascot and X! Tandem, was generated using an in-house script (see methods section for details). CpBase ID, CpBase protein model accession number; peptide, peptide sequence; position, peptide start position as matched to protein sequence; spectra (dimethylated), number of spectra identifying the N-terminally dimethylated form of this peptide; spectra (acetylated), number of spectra identifying the N-terminally acetylated form of this peptide; gene name, CpBase gene name annotation.
(XLS)

Table S7 **List of 63 plastid transit peptides derived from identified mature N termini of nuclear-encoded, plastid-imported *T. pseudonana* proteins.** ID, JGI protein ID (* model manually modified or extended); TP sequence, inferred plastid transit peptide sequence; Met, presence of a Met directly preceding the identified mature N terminus that may be removed after TP cleavage; spectra (acetyl), number of spectra identifying acetylated form of this N terminus; spectra (dm), number of spectra identifying dimethylated form of this N terminus; TP length, transit peptide length; TP charge, calculated net charge of transit peptide; 6 N-term aa, net charge of 6 most N-terminal protein residues.
(XLS)
